# The Role of Heat Shock Protein 70 (HSP70) in the Pathogenesis of Ocular Diseases—Current Literature Review

**DOI:** 10.3390/jcm13133851

**Published:** 2024-06-30

**Authors:** Monika Modrzejewska, Oliwia Zdanowska

**Affiliations:** 1 2nd Department of Ophthalmology, Pomeranian Medical University in Szczecin, Powstańców Wielkopolskich 72, 70-111 Szczecin, Poland; 2 K. Marcinkowski University Hospital, 65-046 Zielona Góra, Poland

**Keywords:** heat shock protein 70 (HSP70), heat shock protein 70 eyes, heat shock protein 70 ocular diseases

## Abstract

Heat shock proteins (HSPs) have been attracting the attention of researchers for many years. HSPs are a family of ubiquitous, well-characterised proteins that are generally regarded as protective multifunctional molecules that are expressed in response to different types of cell stress. Their activity in many organs has been reported, including the heart, brain, and retina. By acting as chaperone proteins, HSPs help to refold denatured proteins. Moreover, HSPs elicit inhibitory activity in apoptotic pathways and inflammation. Heat shock proteins were originally classified into several subfamilies, including the HSP70 family. The aim of this paper is to systematise information from the available literature about the presence of HSP70 in the human eye and its role in the pathogenesis of ocular diseases. HSP70 has been identified in the cornea, lens, and retina of a normal eye. The increased expression and synthesis of HSP70 induced by cell stress has also been demonstrated in eyes with pathologies such as glaucoma, eye cancers, cataracts, scarring of the cornea, ocular toxpoplasmosis, PEX, AMD, RPE, and diabetic retinopathy. Most of the studies cited in this paper confirm the protective role of HSP70. However, little is known about these molecules in the human eye and their role in the pathogenesis of eye diseases. Therefore, understanding the role of HSP70 in the pathophysiology of injuries to the cornea, lens, and retina is essential for the development of new therapies aimed at limiting and/or reversing the processes that cause damage to the eye.

## 1. Introduction

The integrity of proteins is important for the maintenance of cell health. Different types of cells in the eye have specific mechanisms facilitating the correct folding of proteins, the refolding of damaged proteins, or the removal of those with irreversible damage. Heat shock proteins (HSPs), otherwise known as chaperone proteins (chaperones), are a very important factor involved in key intracellular processes [1]. HSPs present in the normal eye are overexpressed in response to various types of cell stress. Moreover, HSPs elicit inhibitory activity in apoptotic pathways and inflammation. The literature also describes the role of HSP70 proteins in regulating cell signalling and modulating the immune response, as well as their involvement in chronic diseases such as diabetes, obesity, and insulin resistance [2]. The role of HSPs as anti-inflammatory mediators is not well understood, although it is likely that they reduce the level of inflammatory mediators such as tumour necrosis factor α (TNF-α), presumably by interacting with the nuclear factor κB (NFκB), a nuclear transcription factor for many genes associated with inflammation, and its inhibitor, IκB [3]. The expression of HSP is induced by the transcription factor Heat Shock Factor 1 (HSF1) [4,5,6,7]. In the chaperone model, HSF1 in cells that are not exposed to stress exists in an inactive complex with HSP90, HSP40, and HSP70. When higher levels of heat shock proteins are required in response to cell stress, HSF1 is released from the complex and migrates to the nucleus. Active homotrimeric, hyperphosphorylated HSF1 binds with Heat Shock Elements (HSEs) in the promoter of *HSP genes*, leading to their upregulation [4,5,7].

Heat shock proteins were originally grouped based on their molecular weight, which ranges from 15 to 110 kDa [2]. Initially, researchers identified four classes of HSP: small HSP, HSP60, HSP70, and HSP90, where the numbers corresponded to the molecular weight in kilodaltons. However, because of the increasing number and the resulting inconsistencies in labelling, a new system with seven classes was adopted: *HSPH* (HSP110), *HSPC* (HSP90), *HSPA* (HSP70), *DNAJ* (HSP40), *HSPB* (small HSP), and chaperone families *HSPD/E* (HSP60/HSP10) and TRiC (TCP-1) [8,9,10].

HSP70 family proteins structurally contain a domain that binds the hydrophobic components of damaged or misfolded polypeptides, thereby preventing their accumulation. This domain is activated through the ATP-dependent N-terminal domain. Hydrolysis of ATP to ADP in the N-terminal domain is facilitated by nucleotide exchange factors, which stimulate protein release. The regulation of protein binding by HSP70 also involves interactions with other proteins, known as co-chaperones or DnaJs, which are responsible for the selective segregation of polypeptides and their attachment to the free domain of HSP-70 [11]. The energy for this process is derived from ATP; the hydrolysis of ATP provides the energy needed for the unfolding of the protein, which is subsequently released. HSP70 expression increases in cells exposed to various forms of cellular stress, aiding in the formation of polypeptides and their optimal spatial organisation. Additionally, HSP70 proteins assist in transporting proteins to specific cellular organelles. The distribution of individual members of the HSP70 family across cell organelles is shown in the table below (Table 1). HSPs can be presented on the cell membrane and released into the extracellular space, leading to detectable levels of HSPs in the bloodstream [10]. HSP proteins can exist both intracellularly and extracellularly, enabling them to perform different functions depending on their location. Intracellularly, they exhibit cytoprotective effects and regulate the degradation of damaged proteins. Extracellularly, they participate in the response of the immune system [12].

## 2. Materials and Methods

The authors reviewed the English-language literature published over the past seven years (2017–2024) and references cited in search engines like PubMed, NIH, and Google Scholar, focusing on the presence of HSP70 in the human eye and its role in the pathogenesis of eye diseases reported to date. The PubMed search engine yielded 86 articles using the keywords “heat shock protein 70 eyes” and 10 articles with “heat shock protein 70 ocular diseases”. Out of a total of 96 articles, after excluding those that were irrelevant, single case reports, and non-English publications, 75 were ultimately selected for this review.

## 3. Discussion

### 3.1. HSP70 in the Normal Eye

The HSP70 (HSPA) family is represented in the human genome by 13 members, including, but not limited to, the constantly expressed HSC70 (*HSPA8*), stress-induced HSP70-1 (*HSPA1A*, also known as *HSP72* and *HSPA1*), and HSP70-2 *(HSPA1B*) [14].

HSP70 has been identified in the cornea, lens, and retina of the normal eye. In the normal cornea, HSP70 functions as an ATP-dependent chaperone protein [15]. When the eye is exposed to cell stress, HSP70 synthesis increases due to the greater amount of misfolded or denatured proteins [16,17]. This process is also observed in the human cornea, where HSP70, as a component of the response to cell stress, inhibits apoptosis or DNA damage and determines cell survival [14]. The presence of HSP70 has also been confirmed in the lens [18]; HSP70 is mainly found in the epithelial and superficial fibres of the lens cortex, where together with HSP40, this protein is located in areas exhibiting significant protein synthesis, where it is involved in protein transport between cellular compartments, protein folding, fibrosis, and regulation of the heat shock response [19], as demonstrated in studies by Xiukun et al. [20]. The amount of HSP40 and HSP70 in the lens decreases with age [18], which can lead to structural changes in the lens that may indirectly contribute to pathologies. HSP27 was found in most areas of the lens, but its amount did not decrease with age [18]. HSP70 was also detected in the human embryonic retina at 20–33 weeks of gestation, which coincides with the formation of nuclear layers. This may suggest that HSP70 plays an important role in the normal development of the retina [21]. Other studies have suggested that HSP70 is necessary for the protection of retinal cells against photic injury [17,22].

### 3.2. The Role of HSP70 in the Pathogenesis of Ocular Diseases

#### 3.2.1. Cornea

One of the most important functions of the cornea is to maintain its clarity. Scarring from wounds in the corneal epithelium can reduce the clarity of the cornea and thus cause visual impairment. When damaged, the remaining corneal epithelium migrates to the injured area in an attempt to repair the defect [23]. Studies have revealed that HSP70 plays a role in the wound healing process of the human cornea, and a fibronectin derivative phosphorylates and increases the expression of HSP70 in human corneal epithelial cells, while a shortage of HSP70 has been found in slowly healing wounds [24]. HSP70 is also an important factor involved in the healing of corneal injuries in rats [25] and other animals [26].

#### 3.2.2. Lens

Structural changes in the lens can contribute to pathologies. Many different causes of cataract have been identified. For example, Banh et al. [27] reported that chaperone proteins HSP70 and HSP90 may have a protective role against epithelial-to-mesenchymal transition in the rat lens induced by TGF-beta (transforming growth factor β). TGF-β is also known to play a key role in pathological fibrosis, including subcapsular cataract formation [28]. These researchers and Dzialoszynski et al. [29] have suggested that under stress conditions, HSP70 may be responsible for the protection of lens clarity. A 2023 study by Shanbagh et al. [19] found that *HspA4*/Hsp70 expression varies with the morphological type of paediatric cataract. *HspA4* levels were significantly lower in posterior subcapsular cataracts compared to post-traumatic, postpartum, or prenatal cataracts. In prenatal cataracts, *HspA4* expression was significantly higher than in secondary and infectious cataracts [19].

#### 3.2.3. Retina

Retinal ischaemia develops when the blood supply is disturbed due to occlusion, injury, or regression of the retinal vessels, or due to increased intraocular pressure. Occlusion of the central retinal artery, retinal veins, glaucoma, traumatic optic neuropathy, or diabetic retinopathy are different forms of ischaemic retinopathy linked with visual impairment and blindness [30,31]. Retinal neurons, particularly retinal ganglion cells (RGCs), are sensitive to ischaemia. All the above-mentioned diseases lead to thinning of the nerve fibre layer as a result of injury or loss of ganglion cells [32,33].

Numerous studies have demonstrated that HSPs, including HSP70, α-crystalline, HSP40, and HSP110, increase the survival of retinal neurons in the model of glaucoma [34,35], optic nerve crush [35], polyglutamine diseases [35,36], autoimmune uveitis [37,38] and retinal detachment [39]. Liu et al. [40] reported transient upregulation of HSF1 after retinal ischemia-reperfusion (IR) injury. Using transgenic mice carrying the full-length human HSF gene, they demonstrated that increased expression of HSF1 induced the expression of HSP70, prevented intracellular stress, decreased tau phosphorylation and attenuated inflammatory response, and had a neuroprotective effect against retinal IR injury. Piri et al. [35] also reported that HSP70 and α-crystallins enhance the survival of RGC by inhibiting many apoptotic pathways and stabilising the cell cytoskeleton or preventing misfolding of proteins, as well as through their involvement in the removal of defective proteins. Despite numerous reports in the literature indicating increased expression and protective functions of HSP70 in various types of retinal diseases, the role of chaperone proteins in these conditions is still not clearly defined. A study by Chidlow et al. [41] examined the expression of HSP70 and HSP27 in models of RGC degeneration by different mechanisms: axonal damage, somato-dendritic damage, chronic hypoperfusion, and experimental glaucoma. However, the study results indicate that HSP70 was not induced in any degeneration model in the retina, optic nerve, or visual cortex, thereby contradicting the presumed protective role of HSP70 for RGCs. These findings suggest that it is not possible, based on previous research, to conclusively determine whether shock proteins have a protective role in retinal diseases, highlighting the need for more extensive research in this area.

**Figure 1:**
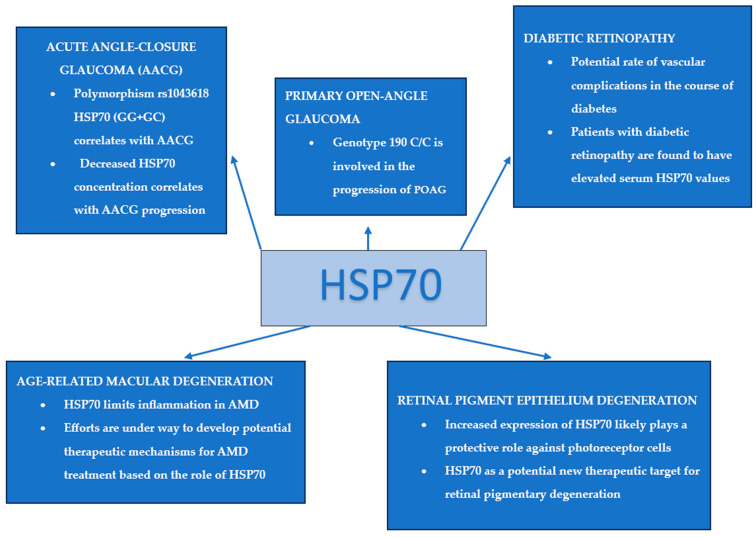
The importance of HSP70 in retinal diseases.

In Figure 1, the authors present the most important literature reports on the role of HSP70 in retinal diseases. Figure 1 is a schematic version of the news contained in this article, graphically illustrating this complex issue, in order to clarify information and better understand the achievements of medicine in this field.

#### 3.2.4. Diabetic Retinopathy

In 2022, a study by Al-Zuhaeri et al. [2] found that HSP70 levels were higher in patients with type 2 diabetes than in healthy controls. Significantly higher levels were observed in patients with poorly controlled diabetes and those with long-term diabetes, which may suggest that HSP70 is involved in disease progression and could be a potential indicator of metabolic disorders and vascular complications of diabetes, including diabetic retinopathy [2]. The literature has discussed the mechanism linking HSP70 with proliferative retinopathy. It has been shown that the formation of complexes between *HSPA*/HSP70 and matrix metallopeptidase 9 (MMP-9) is increased in the retinas of diabetic mice. Under oxidative stress, the activation of MMP-9 leads to mitochondrial abnormalities associated with diabetes, which results in retinal damage. MMP9 inhibitors work by inactivating MMP-9, which, under conditions of increased glucose concentration in the body, binds more strongly to Hsp70/Hsp60 and is transported to the mitochondria, where it exerts destructive effects. These inhibitors prevent the process, thus preserving mitochondrial HSPs, which play a crucial local role in controlling mitochondrial protein formation and exhibit anti-apoptotic effects. Thus, MMP-9 inhibitors may have potential preventive effects in the development of diabetic retinopathy. The authors of this article would like to emphasise that the reports discussed above do not unequivocally define the role of HSP70 in the development of proliferative retinopathy as protective. However, they do show an increased expression of these chaperone proteins under conditions of diabetes-induced oxidative stress and apoptosis. This may suggest a compensatory function aimed at preventing these phenomena. Nevertheless, further studies are necessary to clarify the significance of HSP70 in diabetic retinopathy [42].

#### 3.2.5. Retinal Pigment Epithelium (RPE) Degeneration

Furukawa et al. [43] investigated the role of HSP70 in photoreceptor cell death. Using the N-methyl-N-nitrosourea (MNU)-induced photoreceptor cell death model in mice for analysis, they concluded that the upregulation of intact HSP70 expression by its inducer is likely to protect photoreceptor cells. They also suggested that HSP70 expression inducers, such as valproic acid or geranylgeranylacetone (GGA), could be candidate therapeutic agents for the prevention of retinal degenerative diseases, including retinitis pigmentosa. Moreover, in 2023, Zhang et al. [44] showed that GGA-induced HSP70 expression significantly reduced gliosis, autophagosome accumulation, and apoptosis in retinal I/R (ischemia/reperfusion) damage, indicating that GGA has a protective effect on retinal I/R damage. Furthermore, the protective effect of GGA mechanistically relied on the activation of the PI3K/AKT/mTOR signalling pathway [44].

In 2020, Jiang et al. [45] evaluated the impact of increased HSP70 expression in mutant photoreceptor mouse models of retinal degeneration. A temporary increase in endogenous HSP70 expression at early stages, followed by a significant decrease as cells died, suggests an initial adaptive response to cellular stress. Overexpression of HSP70 has shown varying effects in photoreceptors, depending on the type of mutant protein. In RHOT17M mice, where mutant rhodopsin is abnormally folded, increased HSP70 expression slightly improved photoreceptor survival. However, in rd10 mutants, it led to more severe retinal degeneration. In Rpgrip1−/− mice, which exhibit a ciliary defect, higher HSP70 levels did not affect photoreceptor survival or function. Further research on HSP pathways and associated chaperone networks in photoreceptors is needed to establish therapeutic strategies targeting proteostasis [45].

In 2020, Lyu et al. [46] investigated the effect of leucynostatin on HSP70 expression in stressed canine RPE cells. They showed that leucynostatin can increase HSP70 expression in these cells in response to arsenic-induced stress in a dose-dependent manner. This effect is likely mediated through the activation of heat shock factor-1 (HSF1), suggesting that leucynostatin could potentially serve as a novel co-inducer of HSP70 expression [46].

In 2018, Kern et al. [47] described the effect of thermal exposure on the death kinetics of cultured RPE cells. HSP70 is suggested to be a key factor in the therapeutic effect of hyperthermia treatment in various fields. This study highlights significant differences in the strength of HSP70 induction after sublethal and lethal irradiation, which is not proportionate to the increase in temperature. The findings suggest that RPE cell death after thermal irradiation typically occurs through apoptosis. HSP70 expression induced by thermal irradiation depends on temperature and the death of neighbouring cells, indicating HSP70’s key role in apoptosis and wound healing in RPE cells. The 24–48 h increase in HSP70 highlights its long-term role in cellular responses after both sublethal and lethal thermal laser irradiation. Immunofluorescence of HSP70 revealed that sublethally irradiated cells in the irradiation centre expressed significantly increased amounts of intracellular HSP70. Understanding the role of HSP70 in irradiating RPE cells may help clarify the therapeutic mechanisms of sublethal thermal stimulation of the RPE and the prevention of retinal degenerative diseases [47].

In 2019, Valdés-Sánchez et al. [48] explored the mechanism of RPE degeneration due to PRPF31 mutations and the role of HSP70 in treating this condition. They found that the PRPF31 protein forms cytoplasmic aggregates in the RPE, reducing the protein level of this splicing factor in the nucleus. A haploinsufficient and dominant-negative mechanism is involved in retinal degeneration associated with PRPF31 mutations. Protein aggregation leads to the overexpression of HSP70, which may offer a novel therapeutic target for treating retinal degeneration caused by PRPF31 mutations [48].

In the same year, Yu et al. [49] described a novel mutation of rhodopsin, R135W, which is associated with an autosomal dominant form of retinitis pigmentosa. This mutation induces endoplasmic reticulum stress, protein misfolding, and apoptosis in RPE cells. They demonstrated that HSP70 alleviated RPE stress and prevented R135W rhodopsin-induced apoptosis by reducing abnormal protein folding signalling. These findings highlight the potential therapeutic significance of HSP70 in this form of retinitis pigmentosa [49].

In 2018, Ghaderi et al. [50], in their study, demonstrated the contribution of the GRP78 protein, also called Hsp70-5 or BIP, to the defence/adaptive response of RPE cells exposed to endoplasmic reticulum stress. The study was conducted in a rat model of RPE to which the *HSPA5* gene was transferred via adeno-associated virus. It was concluded that overexpression of this gene stimulated PERK and ATF6α (stress modulators) and activated the proapoptotic cascade. This study demonstrates the protective role of BIP for the survival of RPE cells and points to promising possibilities for the future therapeutic direction of RPE degeneration [50]. In addition, in 2017, Kang et al. [51] evaluated the efficacy of sulforaphane in the treatment of retinitis pigmentosa using mouse models, demonstrating the efficacy of sulforaphane in reducing retinitis pigmentosa by suppressing GRP78 expression, thereby reducing photoreceptor apoptosis [51].

#### 3.2.6. Age-Related Macular Degeneration (AMD)

The pathogenesis of AMD is strongly linked to RPE dysfunction. RPE cells, sensitive to oxidative stress, phagocytose the outer segments of the photoreceptor layer. Due to incomplete breakdown, this contributes to the accumulation of lipofuscin, which significantly increases oxidative stress and inflammatory responses. In response to the ongoing inflammatory process in AMD patients, there is an intense secretion of pro-inflammatory cytokines [52]. In 2019, Yang et al. [53] evaluated cytokine production by RPE cells induced by 4-hydroxynonenal (4-HNE) using a cytokine system. 4-HNE, a lipid peroxidation product, contributes to the inhibition of RPE lysosomal protease activity and enhances lipofuscin synthesis, which in turn induces AMD progression. 4-HNE exerts pro-inflammatory effects by increasing the extracellular release of HSP70. The mechanism of action of various HSP70 inducers, such as Arimoclomol, Paeoniflorin, and methyl-β-cyclodextrin (MBC), was traced. Paeoniflorin showed a weaker anti-inflammatory effect than Arimoclomol, while MBC exhibited a synergistic effect with the other two substances. This study highlights the roles of chaperone proteins in reducing inflammation in AMD and argues for further research to develop therapeutic approaches targeting the mechanisms discussed above [53].

Long-term, excessive oxidative stress in the RPE surpasses the protective capacity of HSP70, leading to retinal degeneration that can cause vision loss. Providing exogenous HSP70 to limit disease progression seems like a promising approach. Kumar et al. [54] addressed this issue in their review, which described HSP70’s role as an immunomodulator, proteolytic pathway gatekeeper, and regulator of apoptosis, calling it a potential therapeutic target for AMD. They highlighted studies that have been published on the therapeutic role of HSP70 in AMD, including the following:

- The hypothesised protective function of the HSP70 co-inducers paeoniflorin, celastrol, leucynostatin, and arimoclomol, which facilitate the transcriptional activation of HSF1.
- Exogenous enhancement of HSP70 levels by delivery of rhHSP70 (recombinant human HSP70), with evidence supporting its antioxidant function reported in the literature [52].
- HSP70-inducing retinal laser therapies [47].

Based on these studies, it has been inferred that HSP70 is close to meeting the criteria of a molecular target for AMD therapy, but more research is needed on its application [54].

#### 3.2.7. Glaucoma

Glaucoma is a common ocular disease associated, among other things, with increased intraocular pressure due to abnormal flow of the aqueous humour. Cao et al. [55] analysed the effects of HSP70 on human trabecular meshwork cell injury induced by UVB and concluded that HSP70 overexpression promoted cell proliferation and inhibited apoptosis. HSP70 was also found to improve cell viability and inhibit trabecular retinal cell apoptosis by inhibiting the Smad pathway. This implies that HSP70 may become a potential therapeutic target in the treatment of glaucoma. Several other studies have demonstrated that HSP or anti-HSP antibodies are involved in the pathogenesis of glaucoma. More intense immunostaining of HSP in glaucomatous eyes may reflect the role of these proteins in the cellular defence mechanism in response to stress or injury [56]. Numerous data suggest that HSPs as chaperones may be critical for the survival of retinal ganglion cells but may also have a pathogenic effect in patients with optic neuropathy associated with glaucoma by stimulating an immune response. Heat shock proteins are identified as target antigens of T-cell responses in glaucomatous mice and humans with glaucoma. In addition, T-cells infiltrating the retina cross-react with human and bacterial HSPs. Mice raised in the absence of commensal microflora do not develop glaucomatous T-cell responses or related neurodegeneration. These studies provide compelling evidence that glaucomatous neurodegeneration is partly mediated by T-cells that are pre-sensitised by exposure to commensal microflora [57]. However, further research is still needed on other polymorphic variants of genes involved in the mechanism of neurodegeneration to better understand the molecular basis of glaucoma and find early diagnostic markers for its development.

##### Primary Open-Angle Glaucoma (POAG)

Previous studies on the expression of the HSP70-1 gene, its polymorphisms, and the role of the HSP70 protein in primary open-angle glaucoma (POAG) do not present a uniform position, and the results are not entirely consistent. A 2014 study by Nowak et al. [58] confirmed the correlation of the 190G/C polymorphism of the HSP70-1 gene with the occurrence of POAG. However, this correlation was dependent on specific clinical parameters, such as the optic disc diameter-to-depression ratio (c/d) *p* = 0.014 and the neuroretinal ring area *p* = 0.024. This finding may indicate the involvement of the 190G/C polymorphism in progressive neurodegeneration of the optic nerve [58]. Another study by Nowak et al. [59] found that *HSPA1A* gene expression in POAG patients increased inversely proportional to the decreasing neuroretinal ring area. The same study also compared *HSPA1A* gene genotypes and demonstrated that the 190 G/C genotype of the HSP70-1 gene occurred with comparable frequency in both healthy individuals and POAG patients, while the 190 C/C genotype was more frequent in patients with an increased nerve fibre index (NFI) in polarimetric nerve fibre layer thickness analysis, confirming the involvement of the 190 C/C genotype in the progression of POAG [59]. In 2018, the levels of HSP70 proteins in the blood of POAG patients were evaluated; however, there was no statistical difference in the levels of these chaperone proteins between patients and a control group of healthy individuals, indicating no association of HSP70 proteins with the development of POAG [60].

##### Acute ANGLE-closure Glaucoma (AACG)

A 2016 meta-analysis by Rong et al. [61] examined the genetic determinants of primary angle-closure glaucoma (PACG) and identified 10 polymorphisms in eight genes that are closely associated with PACG, including the HSP70 polymorphism (rs1043618, GG + GC). In 2019, a correlation between the G + 190C polymorphism in HSP70 and PACG was detected among Pakistani patients; this association was not observed in POAG patients [62]. Additionally, in 2021, Chen et al. [63] found a close correlation between reduced HSP70 levels and the progression of acute angle-closure glaucoma (AACG). HSP70 concentration in patients before treatment was closely related to parameters such as central anterior chamber depth, peripheral anterior chamber depth, anterior angle, and intraocular pressure height, and significantly increased after successful treatment. The authors also proposed HSP70 levels as a promising future indicator for monitoring adverse effects during treatment of AACG patients. The index decreased when adverse effects such as anterior chamber inflammation, choroidal detachment, fibrotic exudation, corneal oedema, or age-related macular degeneration (AMD) occurred [63]. The above studies suggest that HSP70 could be an effective indicator in the future, for both diagnosing and monitoring the progression of the disease. Furthermore, the occurrence of primary angle-closure glaucoma (PACG) may be closely correlated with specific polymorphisms of genes encoding HSP70 proteins. These promising findings encourage further research to understand the exact mechanism of action of HSP70 on acute angle-closure glaucoma (AACG).

#### 3.2.8. Pseudoexfoliation Syndrome

Pseudoexfoliation syndrome (PEX) is a condition belonging to the elastoses. It is characterised by the deposition of microfibrillar material in the tissues of the anterior segment of the eyeball and in many other organs. The incidence of PEX syndrome increases with age. Ocular complications of PEX include keratopathy, anterior chamber hypoxia due to decreased capillary flow, aqueous fluid tyndallization, iris depigmentation, posterior adhesions, difficulty with pharmacologic pupillary dilation, accelerated cataract development, instability of the lens ligament apparatus (iris tremor and lens subluxation), retinal detachment, and secondary glaucoma [64].

In 2020. Güler et al. [65] conducted a study to identify molecules that may be linked to the pathogenesis of PEX syndrome. They measured the levels of HSP70, periostin, and irisin in the aqueous fluid of patients with pseudoexfoliation syndrome and cataracts and compared them with the results of a control group (cataract patients without pseudoexfoliation syndrome). Concentrations of HSP-70, periostin, and irisin in the aqueous fluid of patients with PEX were 1.5 times higher than in the control group without PEX. The exact reasons for this increase are unknown; it is suspected that inflammation and oxidative stress may cause an increase in these substances in PEX patients. This study underscores the need for more detailed research into the significance of these substances in patients with PEX [65].

In 2020. Hayat et al. [66] investigated the epigenetic regulation of HSP70 and its potential role in the pathophysiology of PEX and glaucoma. HSP70 expression levels were significantly reduced in the lens capsule with PEX, but not in glaucoma. Hydrogen sulphite sequencing of the lens capsule of the tested individuals revealed that CpG islands located in the exon region, but not in the HSP70 promoter region, showed hypermethylation only in individuals with PEX. The study observed a corresponding increase in DNA methyltransferase 3A (DNMT3A) expression only in individuals with PEX, suggesting de novo methylation at this stage of the disease state. Treatment of human lens epithelial B-3 (HLE B-3) cells with a DNMT inhibitor restored HSP70 expression after reducing methylation levels at exon CpG sites. We confirmed that reduced HSP70 expression correlates with hypermethylation of CpG HSP70 islands in patients with PEX [66].

#### 3.2.9. Eye Cancers and Autoimmune Diseases

Retinoblastoma (RB) is a malignant retinal tumour that is most common in young children. To find new therapy models, a lot of research has been conducted to explain the molecular background of this cancer. The antiapoptotic effects of HSP on RB formation have not been explained in detail. Jiang et al. [67] detected HSP70 and HSP90 in tumour cells, suggesting the involvement of these proteins in inhibiting apoptosis in RB.

Publications discussing the importance of HSP70 in Behçet’s disease are present in the literature. In 2001, a study by de Smet et al. found elevated levels of anti-HSP70 antibodies in patients with uveitis associated with Behçet’s disease compared to healthy controls [68]. In 2010, Karadağ et al. investigated HSP70 levels in patients with Behçet’s disease and detected elevated values, regardless of disease activity, concluding that HSP70 may play a role in chronic Behçet’s disease [69]. In 2013, Sahebari et al. [70] evaluated serum levels of HSP-70 in patients with Behçet’s disease. The levels were higher in patients compared to healthy controls and increased further when patients had uveitis. Moreover, the study compared results from patients with uveitis due to Behçet’s disease to those with idiopathic uveitis, finding higher HSP70 levels in the former group. The study also examined anti-HSP70 levels in the same group of patients, but found no correlation between anti-HSP70 and Behçet’s uveitis. In conclusion, the aforementioned study suggests that HSP70 may serve as an indicator to differentiate uveitis in Behçet’s disease from uveitis of other etiologies and to predict the occurrence of uveitis in Behçet’s disease [70]. In contrast, a 2018 study by Balkan et al. [71] evaluated the expression of the HSP70 gene and did not corroborate the results of the previous study, finding no significant difference in the expression levels of HSP70 between patients with Behçet’s uveitis and healthy subjects [71]. In summary, although most studies to date suggest a correlation between HSP70 levels and Behçet’s disease, particularly when complicated by uveitis, inconsistencies across different studies remain. Therefore, to determine whether HSP70 family proteins have predictive value in this disease entity, further studies are required.

#### 3.2.10. Ocular Toxoplasmosis

In 2020, Chiquet et al. [72] investigated the role of IgG class antibodies, specifically anti-HSP70.1, in serum and aqueous fluid for diagnosing ocular toxoplasmosis. They found that the concentration of anti-HSP70.1 antibodies was not significantly different in the patient group (those with active uveitis) compared to the control group, indicating that it was not a useful diagnostic indicator of ocular toxoplasmosis. However, they did find a strong correlation between the antibody levels and retinal damage in the patient group [72].

In 2021, Mitra et al. [73] published a paper showing that T. gondii infection increases host HSP70 expression. Additionally, the HSP70 inhibitor 2-phenylethylsulfonamide (PES) affects the chaperone function of HSP70, leading to a disruption of host autophagy and a reduction in the intracellular proliferation of T. gondii. PES also targets the HSP70 homolog in the parasite itself, suggesting the potential for dual targeting of both host and parasite proteins with a single molecule. This insight may be useful in therapeutic design [73].

The authors summarised the results of the cited studies in a table (Table 2) to better clarify the literature discussed above.

## 4. Conclusions

This HSP70 is present in the eye under normal conditions, and is overexpressed in response to various types of cell stress. HSP70 has been identified in the cornea, lens, and retina of a normal eye, where it inhibits apoptotic pathways and inflammation, acting as a chaperone to prevent degenerative changes and protect healthy cells from damage. The increased expression and synthesis of HSP70 induced by cell stress has also been demonstrated in eyes with pathologies such as glaucoma, eye cancers, cataracts, scarring of the cornea, ocular toxpoplasmosis, PEX, AMD, RPE, or diabetic retinopathy. Most of the studies cited in this paper confirm the protective role of HSP70. However, there are still limited data on the role of heat shock proteins in the pathogenesis of ocular diseases. Further, more extensive research is required for a better understanding of the molecular mechanisms regulating injuries in the cornea, lens, and retina, important for developing new therapies aimed at reducing visual impairment and/or restoration of vision.

## Figures and Tables

**Table 1:** Intracellular localisation of HSP70 family members [13].

Gene	Encoded Protein	Synonyms	Intracellular Location
*HSPA1L*	Hsp70-1L	HSP70-1L, HSP70-HOM, HSP70T, hum70t	cytosol, nucleus
*HSPA1A*	Hsp70-1	HEL-S-103, HSP70, HSP70-1, HSP70-1A, HSP70-2, HSP70.1, HSP70.2, HSP70I, HSP72, HSPA1	cytosol, nucleus
*HSPA1B*	Hsp70-2	HSP70-1, HSP70-1B, HSP70-2, HSP70.1, HSP70.2, HSP72, HSPA1, HSX70	cytosol, nucleus
*HSPA2*	HSPA2	HSP70-2, HSP70-3	cytosol, nucleus
*HSPA5*	Hsp70-5	BIP, GRP78, HEL-S-89n	endoplasmic reticulum
*HSPA6*	Hsp70-6	HSP70B’	cytosol
*HSPA7*	Hsp70-7	Hsp70B	_
*HSPA8*	Hsp70-8	HEL-33, HEL-S-72p, HSC54, HSC70, HSC71, HSP71, HSP73, HSPA10, LAP-1, LAP1, NIP71	cytosol, lysosomes
*HSPA9*	Hsp70-9	CSA, MOT; MOT2, SAAN, CRP40, EVPLS, GRP75, PBP74, GRP-75, HSPA9B, SIDBA4, MTHSP75, HEL-S-124m	mitochondria
*HSPA12A*	Hsp70-12A	_	_
*HSPA12B*	Hsp70-12B	_	_
*HSPA13*	Hsp70-13	STCH	endoplasmic reticulum
*HSPA14*	Hsp70-14	HSP70-4, HSP70L1, MSANTD7	cytosol

**Table 2:** The role of HSP70 in the pathogenesis of ocular diseases—summary of the main conclusions of the cited studies.

Disease	Authors	Year of Publication	Main Conclusions
Keratopathy	Ko et al. [24]	2008	HSP70 plays a role in human corneal wound healing.
Cataract	Dzialoszynski et al. [29]	2016	HSP70 may be responsible for protecting the transparency of the lens.
	Shanbagh et al. [19]	2023	HspA4/Hsp70 expression varies depending on the morphological type of paediatric cataracts.
Diabetic retinopathy	Sayed et al. [74]	2016	The determined serum level of HSP70 in patients with diabetic retinopathy was significantly higher than in healthy patients. The increase in HSP70 levels was consistent, independent of the stage of retinopathy.
	Kowluru et al. [42]	2021	MMP-9 inhibitors may have a potential preventive effect in the development of diabetic retinopathy, preventing the decline of mitochondrial HSPs.
	Al-Zuhaeri et al. [2]	2022	The level of HSP70 was significantly higher in patients with type 2 diabetes compared to controls; with the presence of vascular complications such as proliferative retinopathy, HSP70 levels reached even higher values.
Retinal pigment epithelium (RPE) degeneration	Furukawa et al. [43]	2016	Increasing HSP70 expression likely protects photoreceptor cells. Inducers of HSP70 expression, such as valproic acid and geranylgeranylacetone, may serve as potential therapeutic agents for the prevention of retinal degenerative diseases.
	Kern et al. [47]	2018	HSP70 is an important therapeutic factor in hyperthermia treatment; HSP70 plays a key role in apoptosis and wound healing in RPE cells.
	Valdés-Sánchez et al. [48]	2019	HSP70 is a potential new therapeutic target for the treatment of retinal degeneration caused by PRPF31 mutations.
	Yu et al. [49]	2019	HSP70 is a potential new therapeutic target for an autosomal dominant form of retinal pigmentary degeneration associated with a novel R135W rhodopsin mutation. HSP70 alleviates RPE stress and prevents R135W rhodopsin-induced apoptosis.
	Jiang et al. [45]	2020	Overexpression of HSP70 has been shown to have varying effects on photoreceptors, depending on the type of mutant protein; it can either improve photoreceptor survival or exacerbate retinal degeneration.
	Lyu et al. [46]	2020	Leucinostat increases HSP70 expression in canine RPE cells in response to stress and could potentially serve as a novel co-inducer of HSP70 expression.
	Zhang et al. [44]	2023	Geranylgeranylacetone-induced expression of HSP70 significantly reduces gliosis, autophagosome accumulation, and apoptosis in retinal ischemia-reperfusion injury.
Age-related macular degeneration (AMD)	Yang et al. [53]	2019	The study confirmed the roles of chaperone proteins in reducing inflammation in AMD and investigated the mechanisms of action of three HSP-70 inducers: Arimoclomol, Paeoniflorin, and methyl-β-cyclodextrin, all of which exhibited potential anti-inflammatory effects.
	Kumar et al. [54]	2020	In their review, the authors described potential therapeutic mechanisms based on the role of HSP70 in the treatment of AMD.
Primary open-angle glaucoma (POAG)	Nowak et al. [58]	2014	The correlation of the 190G/C polymorphism of the HSP70-1 gene with the occurrence of POAG was described.
	Nowak et al. [59]	2015	The study noted the involvement of the 190 C/C genotype in the progression of POAG. The 190 G/C genotype of the HSP70-1 gene occurred in this study with comparable frequency in both healthy and POAG patients.
	Nowak et al. [60]	2018	There was no difference in HSP70 levels between patients and controls, indicating no correlation between HSP70 and the development of POAG.
Acute angle-closure glaucoma (AACG)	Rong et al. [61]	2016	The rs1043618 HSP70 (GG + GC) polymorphism correlates with primary angle-closure glaucoma.
	Chen et al. [63]	2021	The correlation of reduced HSP70 levels with the progression of AACG was proven.
Pseudoexfoliation syndrome (PEX)	Güler et al. [65]	2020	In the study, an increase in HSP70 levels was noted in patients with PEX detected in the aqueous humour.
	Hayat et al. [66]	2020	The study observed a decrease in HSP70 expression in the lens capsule of PEX patients; this reduction in expression was associated with hypermethylation of CpG islands.
Retinoblastoma	Jiang et al. [67]	2008	HSP70 was detected in cancer cells, suggesting its involvement in inhibiting apoptosis in retinoblastoma.
Behçet’s Uveitis	de Smet et al. [68]	2001	Elevated levels of anti-HSP70 antibodies were detected in patients with Behçet’s Uveitis compared to healthy controls.
	Karadağ et al. [69]	2010	Regardless of disease activity, elevated levels of HSP70 were detected in the serum of patients with Behcet’s disease.
	Sahebari et al. [70]	2013	In the study, patients with Behçet’s disease had higher levels of HSP70 compared to the control group, with the highest values recorded in patients with Behçet’s Uveitis.
	Balkan et al. [71]	2018	There was no difference in HSP70 levels between patients with Behçet’s Uveitis and healthy controls.
Ocular toxoplasmosis	Chiquet et al. [72]	2020	There was no difference in anti-HSP70 antibody levels between patients with toxoplasmosis uveitis and healthy controls. A correlation between anti-HSP70 antibody levels and retinal damage in patients with ocular tuberculosis was observed.
	Mitra et al. [73]	2021	It was concluded that T. gondii infection increases host HSP70 expression, and the HSP70 inhibitor, 2-phenylethylsulfonamide (PES), was suggested as a potential therapy for toxoplasmosis.

## Data Availability

No new data were created or analysed in this review. Data sharing does not apply to this article.
